# A Study on the Epidemiological-Molecular Role of *Staphylococcus aureus* Strains in the Development of Ventilator-Associated Pneumonia in a Tertiary Hospital in Brazil

**DOI:** 10.3390/antibiotics12081336

**Published:** 2023-08-18

**Authors:** Mariana Fávero Bonesso, Carlos Magno Castelo Branco Fortaleza, Ricardo de Souza Cavalcante, Moises Teixeira Sobrinho, Carlos Fernando Ronchi, Lígia Maria Abraão, Hwang-Soo Joo, Michael Otto, Maria de Lourdes Ribeiro de Souza da Cunha

**Affiliations:** 1 Department of Infectology, Dermatology, Diagnostic Imaging and Radiotherapy, Medical School (FMB) of Sao Paulo State University (UNESP), Botucatu 18618-970, Brazil; carlos.fortaleza@unesp.br (C.M.C.B.F.); ricardo.cavalcante@unesp.br (R.d.S.C.); 2 Department of Chemical and Biological Sciences, Biosciences Institute, UNESP—Universidade Estadual Paulista, Botucatu 18618-691, Brazil; 3 Pathogen Molecular Genetics Section, Laboratory of Bacteriology, National Institute of Allergy and Infectious Diseases, U.S. National Institutes of Health, Bethesda, MD 20814, USA; hwangsoojoo27@duksung.ac.kr (H.-S.J.); motto@niaid.nih.gov (M.O.); 4 Hospital das Clínicas, Botucatu Medical School, University of Sao Paulo State, Botucatu 18618-687, Brazil; moisesteixeirasobrinho@gmail.com (M.T.S.); fernando.ronchi@ufu.br (C.F.R.); 5 Nursing Research and Care Practices, Hospital Samaritano Higienópolis, São Paulo 01232-010, Brazil; ligia.abraao@americasmed.com.br

**Keywords:** *Staphylococcus aureus*, ventilator-associated pneumonia, virulence factors

## Abstract

This study aimed to explore the molecular epidemiology of *Staphylococcus aureus* isolated from patients on mechanical ventilation and the participation of virulence factors in the development of ventilator-associated pneumonia (VAP). A prospective cohort study was conducted on patients under mechanical ventilation, with periodic visits for the collection of tracheal aspirates and clinical data. The *S. aureus* isolates were analyzed regarding resistance profile, virulence, expression of protein A and alpha-toxin using Western blot, clonal profile using PFGE, sequence type using MLST, and characterization and quantification of phenol-soluble modulins. Among the 270 patients in the study, 51 *S. aureus* strains were isolated from 47 patients. The incidence density of *S. aureus* and MRSA VAP was 2.35/1000 and 1.96/1000 ventilator days, respectively; of these, 45% (n = 5) were resistant to oxacillin, with 100% (n = 5) harboring SCC*mec* types II and IV. The most frequent among the tested virulence factors were *ica*A, *hla*, and *hld*. The clonal profile showed a predominance of sequence types originating from the community. Risk factors for VAP were the presence of solid tumors and the *sea* gene. In conclusion, patient-related risk factors, together with microbiological factors, are involved in the development of *S. aureus* VAP, which is caused by the patient’s own strains.

## 1. Introduction

Ventilator-associated pneumonia (VAP) is a type of nosocomial pneumonia that affects 9 to 27% of patients subjected to mechanical ventilation [1,2]. Mortality attributed to VAP varies according to the risk group studied, with surgical and long-stay patients being more likely to die from the disease [3]. Thus, the epidemiological and molecular study of factors related to the development of VAP is of great importance since it can help identify risk groups and develop new interventions.

The most common etiological agents of VAP are aerobic Gram-positive bacteria such as *Staphylococcus aureus* and Gram-negative bacteria such as *Pseudomonas aeruginosa*, *Acinetobacter baumannii*, and *Klebsiella pneumoniae*. *S. aureus* is the main causative agent of nosocomial pneumonia and the second most common agent associated with all nosocomial infections [4,5].

Pneumonias caused by methicillin-resistant *S. aureus* (MRSA) are generally difficult to treat and are associated with high rates of treatment failure [6]. MRSA strains carry the *mec*A gene, which confers resistance to beta-lactam antibiotics and is located on a mobile genetic element (staphylococcal cassette chromosome *mec*—SCC*mec*) of variable size and genetic composition. SCC*mec* typing is widely applied in epidemiological studies and can be useful for clinical purposes. There are currently 14 SCC*mec* types (I to XIV) [7]. Regarding their epidemiological distribution, types I to III are mostly found in hospitals, and the other types are predominantly community-associated. Haque et al. [6] found that SCC*mec* type II in conjunction with host factors is associated with mortality, high vancomycin minimum inhibitory concentrations (MIC), and clinical treatment failure in patients with nosocomial pneumonia caused by MRSA. This finding highlights the importance of epidemiological–molecular research on these microorganisms associated with VAP.

*Staphylococcus aureus* has a great ability to invade host tissues due to the production of different virulence factors. Several studies have addressed the role that each virulence factor plays in the severity of pneumonia and/or lung injury, which range from the investigation of bacterial cytotoxicity [6,8] to exploring the role of each virulence gene. Among the virulence factors that play an important role in pneumonia, alpha-hemolysin or alpha-toxin (Hla) is one of the most prominent cytotoxins that target erythrocytes, epithelial and endothelial cells, T cells, neutrophils, monocytes, and macrophages. Alpha-toxin is an amphipathic, water-soluble, pore-forming molecule that binds primarily to lipids present on the host cell membrane [9,10]. The main function of alpha-toxin is the causing osmotic swelling, rupture, lysis, and subsequently, cell death. It is highly inflammatory, causing pulmonary congestion [11].

Other factors, such as staphylococcal enterotoxins (SEs) and toxic shock syndrome toxin 1 (TSST-1), are superantigens involved in diverse human infections. To explore their participation in pneumonia, experimental studies using rabbits have shown that intrapulmonary instillation of SEB and SEC induces hemorrhage in the pulmonary tissue, causing symptoms of respiratory distress and lethal toxic shock syndrome. The administration of strains carrying the genes for the same enterotoxins resulted in pulmonary pathology and lethality similar to the group that received the purified toxins [12,13].

The participation of Panton–Valentine leukocidin (PVL) in pneumonia has been extensively reported in the literature, especially in pneumonia caused by community-acquired *S. aureus* strains [14,15]. PVL is a pore-forming toxin that targets neutrophils and mitochondria. It induces cell lysis, thereby releasing proinflammatory agents into the extracellular environment that cause tissue necrosis. When present in the lung, PVL is associated with necrotizing pneumonia [16]. Some strains that carry the PVL gene also harbor genes encoding exfoliative toxins (*eta*, *etb*, or *etd*) [17]. The role of exfoliative toxins in skin infections is well understood, but little is known about their participation in pneumonia; hence, research on *S. aureus*-associated pneumonia is needed. 

Another important virulence factor in the pathogenesis of *S. aureus* pneumonia is biofilm formation, particularly in the area of the endotracheal tube. The *icaADBC-*mediated polysaccharide production is an important mechanism for biofilm formation and contributes to the early growth of bacteria [18]. The *bap* gene of *S. aureus* encodes a surface protein Bap (biofilm-associated protein) containing 2276 amino acids. Bap was identified as the main determinant of successful surface adhesion and intercellular adhesion during biofilm formation [18].

In addition to bacterial factors, the involvement of host-related factors in the development of VAP has been widely studied. The use of antimicrobials and antacids, opting for tracheostomy, supine position, parenteral nutrition, AIDS, male gender, lung disease, coma, and trauma are risk factors for the development of VAP [19].

In view of the above considerations, our study aimed to explore the molecular epidemiology of *S. aureus* isolated from patients on mechanical ventilation and to investigate the participation of virulence and patient-related factors in the development of VAP.

## 2. Results

### 2.1. Microbial Isolation and Identification

Among 270 patients seen over the study period (November 2011 to August 2013), 51 *S. aureus* strains were isolated from 47 patients. Eleven strains were recovered from patients with VAP, and more than one isolate was identified in two patients, totaling thirteen *S. aureus* isolates, including 55% methicillin-susceptible *S. aureus* (MSSA) and 45% MRSA. The total incidence density of VAP was 12.9/1000 ventilator days, with 4.31/1000 ventilator days being caused by *S. aureus* (2.35/1000 ventilator days for MSSA and 1.96/1000 ventilator days for MRSA), 1.17/1000 ventilator days by *Acinetobacter baumannii*, 1.17/1000 ventilator days by *Enterobacter* spp., 0.78/1000 ventilator days by *Klebsiella pneumoniae*, 0.78/1000 ventilator days by *Proteus mirabilis*, and 1.56/1000 ventilator days by other agents. Identification of the etiological agent was not possible in some cases of VAP, which corresponded to an incidence density of 2.74/1000 ventilator days.

### 2.2. Detection of mecA Gene, Cassette Chromosome Typing and Antimicrobial Resistance

The *mec*A gene was detected in 45% (23/51) *S. aureus* isolates, 21% (5/23) isolated from patients with VAP. The most common cassette type among all isolates was SCC*mec* type II (n = 18; 78%), followed by type IV (n = 4; 17%) and type I (n = 1; 4%). Among the *S. aureus* isolates associated with VAP, two carried SCC*mec* type II and three carried SCC*mec* type IV.

Determination of MIC in the *S. aureus* isolates using the E-test revealed that none of the isolates had vancomycin MIC higher than 1.5 µg/mL. All isolates were susceptible to quinupristin-dalfopristin, tigecycline, linezolid, and daptomycin by the E-test. The disk diffusion method showed that 81% of the isolates were resistant to penicillin, 58% to erythromycin, 54% to clindamycin, 39% to oxacillin, and 39% to cefoxitin; 13% were positive in the D test. Among the *mec*A gene-positive isolates, 78% (18/23) were also resistant to erythromycin and clindamycin, showing a multidrug resistance profile. None of the MRSA strains were positive in the D test. All isolates were susceptible to gentamicin. Table 1 shows the MIC50 and MIC90 of the drugs. The data sheet with all the data for all 51 *S. aureus* strains isolated in the study can be seen in the Supplementary Materials Table S1.

### 2.3. Virulence Factors of S. aureus Isolates

Among the virulence factors studied, genes associated with biofilm formation (*icaA*) and hemolysins *hla* and *hld* were the most frequent, followed by the *icaD* and *hlb* genes (Table 2). There was no statistically significant association between the presence of the *mec*A gene and virulence factors. A slightly higher frequency of the *sea*, *sec*, *icaC*, and *hlb* genes was observed in *mec*A gene-positive isolates compared to negative isolates, but the association was not statistically significant.

### 2.4. Clonal Characterization of S. aureus Isolates

**Figure 1:**
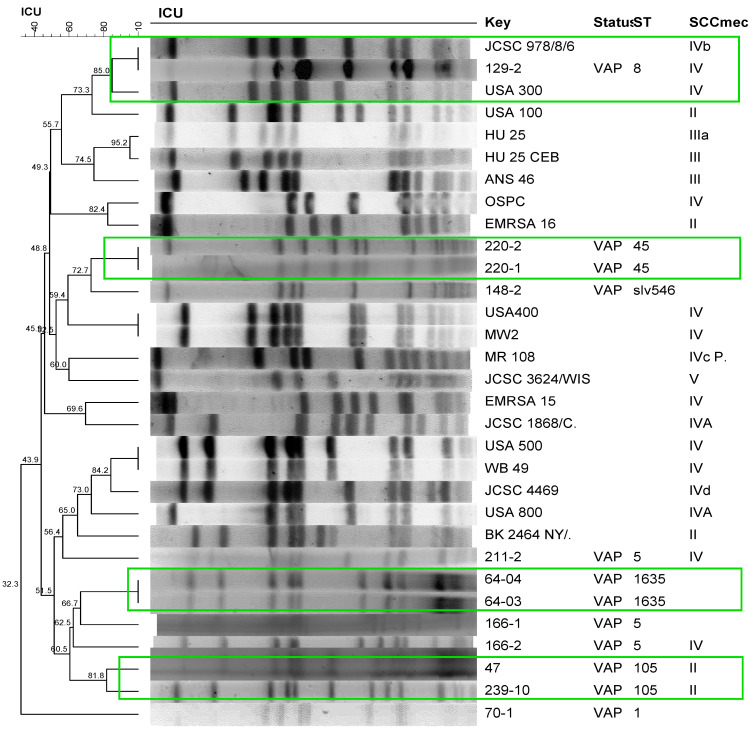
Dendrogram of the PFGE of *S. aureus* strains isolated from patients with ventilator-associated pneumonia (VAP) generated by Dice analysis/UPGMA (BioNumerics, Applied Maths) and their molecular characterization by SCC*mec* typing and MLST. Green frame: Isolates showing >80% similarity after digestion with *Sma*I. International clones used as controls: JCSC 978/8/6, USA300, USA100, HU25, HU25CEB, ANS46, OSPC, EMRSA16, USA400, MW2, MR108, JCSC 3624/WIS, EMRSA15, JCSC1868/C, USA500, WB49, JCSC4469, USA800, BK2464NY/.

Clonal profile analysis of *S. aureus* isolated from patients with VAP using PFGE revealed a polyclonal profile (Figure 1). The most frequent STs were ST105, ST5, and ST398. The only strains that formed clusters were those isolated from the same patient during a period prior to the development of pneumonia, with the exception of one patient (166), who exhibited a different profile in the second sampling in which the isolate carried SCC*mec* IV but belonged to the same ST5 as the first isolate (Figure 1). Comparison of these isolates with international reference strains revealed the clustering of one *S. aureus* strain isolated in this study (129-2) with the international clone USA 300, with 85% similarity. Two isolates (47 and 239-10) formed a cluster with 81.8% similarity, which belonged to the same ST105 and harbored SCC*mec* II. The molecular characteristics, detection, and quantification of phenol-soluble modulins, alpha-toxin, and protein A of the isolates are shown in Table 3 and Table 4.

### 2.5. Risk Factors

The univariate Poisson regression model for analysis of the association of host and microbiological factors with the development of *S. aureus* VAP revealed a positive association with the *sea* and *icaC* genes and a negative association with central nervous system disease. However, in multivariate analysis, the only independent factors that were positively associated with *S. aureus* VAP were the presence of the *sea* gene and the presence of a solid tumor (Table 5).

Regarding the death of patients with VAP caused by *S. aureus* as an outcome, the univariate Poisson model revealed a positive association between liver disease and the presence of the *sea* gene, while the presence of a central venous catheter was negatively associated. However, in multivariate analysis, the presence of the *sea* and *hlb* genes were the only independent factors that were positively associated with death (Table 6).

## 3. Discussion

Nosocomial pneumonias, together with surgical site infections, are the main types of nosocomial infection, and *S. aureus* is the leading associated etiological agent, according to a survey conducted in the United States [4]. The present study showed an ID of *S. aureus* VAP of 2.35/1000 ventilator days, a value higher than that reported in the study by Lee et al. [20] in which the ID of VAP was 1.4/1000 ventilator days and *S. aureus* was the most frequently isolated microorganism.

In our study, the presence of the *mec*A gene, oxacillin resistance, or SCC*mec* type was not associated with the development of VAP or death. Some studies have shown an association between infection with MRSA methicillin-resistant strains and a longer ICU stay and higher mortality rates compared to patients who develop VAP caused by MSSA [21,22]. However, other studies found that methicillin resistance alone was not associated with recurrence, severity, or mortality in *S. aureus* VAP [23,24]. Thus, methicillin resistance does not seem to interfere with the mortality rate in VAP, a fact that raises speculation about whether other factors present in nosocomial strains may contribute to mortality. Interestingly, the frequency of the *icaC*, *sec*, *sea*, and *hlb* genes was slightly higher in *mec*A gene-positive isolates; however, there was no statistically significant association between the simultaneous presence of the *mec*A gene and virulence genes, with MSSA and MRSA isolates showing the same level of virulence.

The wide variability in the clonal profiles among strains isolated from patients with VAP and the formation of clusters only for isolates from the same individual suggest that these isolates are endogenous and that their development occurs from strains that already colonize the patient. Only two isolates (47 and 239) that belonged to the same clone formed a cluster. Previous studies have shown that the pattern of microorganism colonization varies. Gram-positive bacteria colonize the trachea within the first 24 h of mechanical ventilation and are later replaced with Gram-negative bacteria and yeasts [25,26].

Sequence types characteristic of community settings were the types most frequently isolated from patients who developed VAP (ST45, ST5, ST8, ST1635, ST398, and SLV 546), with the exception of nosocomial clone ST105 carrying SCC*mec* II. One strain was grouped with USA300, which belonged to the same ST8 carrying SCC*mec* IV, and was negative for the PVL (*lukSF*-PV) genes. Classic studies of infection with USA300 mainly report necrotizing pneumonia [27] in community-dwelling healthy individuals without traditional factors for MRSA contraction [28]. Despite reports of pneumonia in community-dwelling patients, Pasquale et al. [29] recently described the involvement of USA300 in nosocomial pneumonia. In these cases, participation of this strain is of great clinical importance since, in addition to the difficulty in selecting antimicrobials for treatment, the strain can carry a wide range of virulence factors that can also influence the outcome [3,9]. 

The involvement of STs characteristic of community settings in nosocomial infections has been reported, especially an association of ST8 with outbreaks of difficult-to-treat infections [30]. This observation highlights the need for controlling the transmission of these community-associated strains among patients admitted to the same ICU.

Studies have discussed the participation of virulence factors in the development of VAP in an attempt to identify a marker that permits predicting the development and severity of these infections. The participation of a single virulence factor such as alpha-hemolysin or protein A has been reported to be important, if not crucial, for the development of pneumonia [9,31].

A previous study also found that patients with cancer, especially lung cancer, are at increased risk of developing VAP when compared to other patients [32]. The presence of *sea* in the lungs can elicit an inflammatory response through the production of IFN-γ by CD8 T cells, which causes lesions characteristic of acute respiratory stress syndrome [33]. This finding suggests that strains producing this enterotoxin pose a risk for developing pneumonia associated with severe symptoms.

The only independent factors associated with death were the presence of the *sea* and *hlb* genes. Among the four *S. aureus* strains isolated from patients with VAP who died from this condition, three carried the *hlb* gene and the other the *sea* gene. The presence of enterotoxin A has been linked to aggressive infection [33]. Similarly, the presence of *hlb* in lung tissue can increase neutrophil infiltration by modulating host factors, which contributes to the development of pneumonia [34]. Both death-related factors are known to activate an intense immune response that contributes to breathing problems and can quickly lead to the patient’s death.

The association between the presence of virulence genes and the development of VAP has been little explored, and studies are limited to the analysis of factors such as strain cytotoxicity [8] or few virulence factors in animal experiments [6,31], which offers an excellent perspective of the virulence factors studied. However, our study provides a practical view of virulence factors and new perspectives on the association of microbiological factors with VAP. Within this context, we found that the etiology of VAP is multifactorial, including microbiological factors and risk factors of the host itself.

The main strength of this study is the fact that the patients were followed up since intubation, which allows microbiological study and the analysis of clinical data throughout the period of development of VAP. The results suggest that, in patients admitted to the ICU, VAP can be caused by *S. aureus* strains that already colonize these patients. This suggestion is supported by the STs identified, which are characteristic of strains found in the community that carry important virulence factors involved in the development of VAP and subsequent death of the patient. The identification of patients with VAP previously colonized with *S. aureus* suggests that some type of screening may be useful in predicting the occurrence of infection. However, further studies are still needed to evaluate the best screening approach and its cost-effectiveness. In view of the high mortality associated with VAP, the results of this study also highlight the need for future studies that assess whether *S. aureus* decolonization of known carriers will affect the incidence of VAP.

## 4. Materials and Methods

### 4.1. Study Place and Sample

The study was conducted in two Specialized Intensive Care Units (ICU) of the University Hospital of the Botucatu Medical School (HC-FMB), with 16 and 9 beds, respectively, which admit adult clinical and surgical patients. Patients meeting the following criteria were included: patients undergoing mechanical ventilation started within 48 h of admission to the ICU or during their stay without a previous diagnosis of pneumonia whose families agreed to their participation in the study by signing the free informed consent form.

This is a prospective cohort study whose primary outcomes are VAP caused by *S. aureus* and death due to VAP. The subjects were followed up for a maximum period of eight weeks of ICU stay, twice weekly (Mondays and Thursdays), at an interval of 3.5 days. Clinical data and tracheal aspirates were collected during the evaluations. A doctor of the Committee for the Control of Healthcare-Related Infection (CCIRAS) made the diagnosis of VAP. The criteria recommended by the National Healthcare Safety Network (NHSN) of the Centers for Disease Control and Prevention (CDC) of the United States were used [35].

### 4.2. Collection of Clinical Data 

The following data were obtained during the visit for patient inclusion: gender, age, underlying diseases and comorbidities, length of hospital stay prior to the current hospitalization, procedures, and presence of invasive devices (central venous catheters, indwelling urinary catheters, nasogastric tubes/nasoenteral tubes, and drains). Data comprising the two weeks preceding mechanical ventilation were also collected: past and current use of antimicrobials, history of infectious complications, and results of microbiological tests.

Data on length of stay, procedures, invasive devices, use of antimicrobials, infectious complications, and results of microbiological tests were also collected during the follow-up assessments.

### 4.3. Collection, Isolation, and Microbial Identification

Tracheal aspirate was collected by a physical therapist, doctor, or nurse and immediately sent to the Laboratory of Bacteriology. The clinical material was diluted 1:1 in 1% N-acetyl-L-cysteine (mucolytic), homogenized, and kept for 15 min at room temperature. A 0.1-mL aliquot of this solution was diluted in 9.9 mL sterile saline and seeded with a 0.01-mL calibrated loop onto sheep blood and Baird–Parker agar plates. After incubation for 24 h at 37 °C, the number of colony-forming units (CFU) was determined by multiplying the number of colonies by the corresponding dilution [36]. The cultures were classified as positive when the count was 10^6^ CFU or higher. Microorganisms that grew in the culture medium were subjected to Gram staining for assessment of purity, observation of morphology, and specific staining. After confirmation of these features (Gram-positive cocci in clusters), the catalase and coagulase tube tests were performed according to Koneman et al. [37] for phenotypic identification of *S. aureus*. Catalase- and coagulase-positive isolates were subjected to DNA extraction (see item 4.6) for genotypic identification of *S. aureus* with PCR amplification of the *Sa442* DNA fragment, which is specific for *S. aureus*, following the protocol described by Martineau et al. [38]. After the species was confirmed, the isolates were stored in nutrient broth with glycerol at −70 °C.

### 4.4. Antimicrobial Susceptibility Testing

The antimicrobial susceptibility/resistance profile of all *S. aureus* strains isolated in this study was evaluated using the Kirby–Bauer disk diffusion method using disks impregnated with gentamicin (10 µg), linezolid (30 µg), penicillin G (10 IU), erythromycin (15 µg), and clindamycin (2 µg), as well as the D test for identifying inducible clindamycin resistance, according to the criteria of the Clinical and Laboratory Standards Institute [39]. Methicillin susceptibility was determined using cefoxitin (30 µg) and oxacillin disks (1 µg).

### 4.5. Determination of Minimum Inhibitory Concentration (MIC)

The in vitro MIC of all *S. aureus* isolates against oxacillin, vancomycin, linezolid, daptomycin, quinupristin/dalfopristin, and tigecycline was determined with the E-test. The MIC results are expressed as the proportion of isolates susceptible to each drug according to the CLSI definition [39]. Isolates with intermediate values were classified as resistant.

### 4.6. DNA Extraction from S. aureus

Nucleic acid was extracted from all *S. aureus* strains isolated in the study. The isolates were cultured on blood agar, inoculated individually into BHI broth, and incubated for 24 h at 37 °C. Extraction was performed with the Illustra kit (GE Healthcare) according to the manufacturer’s instructions, and the extracted DNA was stored at −20 °C.

### 4.7. Detection of the mecA Gene

The parameters and primers described by Murakami et al. [40] were used for the detection of the *mec*A gene in the *S. aureus* isolates. International reference strains were included in the assays as positive (*S. aureus* ATCC 33591) and negative control (*S. aureus* ATCC 25923).

### 4.8. Classification of SCCmec in S. aureus

All MRSA isolates were subjected to SCC*mec* typing by multiplex PCR using the primers and parameters described by Milheiriço et al. [36]. The following strains were used as a control for SCC*mec* typing: COL for SCC*mec* type I; N315 for SCC*mec* type IA; PER34 for SCC*mec* type II; AN546 for SCC*mec* type III; HU25 for SCC*mec* type IIIA; and MW2 for SCC*mec* type IV.

### 4.9. Detection of Genes Encoding Virulence Factors 

The genes for the following virulence factors were investigated in all *S. aureus* isolates with PCR using the primers and parameters described in Table 7: PVL (*lukS*-*lukF*-PV); toxic shock syndrome toxin (*tst*); staphylococcal enterotoxins A to C (*sea*, *seb*, and *sec*); biofilm (*icaA*, *icaD*, and *bap*); exfoliative toxins A and B (*eta* and *etb*); and hemolysins α, β, and δ (*hla*, *hlb*, and *hld*). International reference strains were included in all reactions as positive and negative control (Table 7).

### 4.10. Visualization of Amplified Products

The efficiency of the amplifications was monitored with electrophoresis on 2% agarose gel prepared in 1X TBE buffer and stained with SYBR Safe. The size of the amplified products was compared to 100-bp molecular markers, and the gel was photographed under UV transillumination.

### 4.11. Characterization and Quantification of Phenol-Soluble Modulins (PSM) with High-Performance Liquid Chromatography (HPLC)

For the detection and quantification of PSM, 10 µL of an overnight culture was incubated in 1 mL tryptic soy broth for 16 to 18 h, centrifuged at 14,000 rpm for 15 min at 4 °C, and 300 µL of the supernatant was reserved for reversed-phase HPLC-mass spectrometry as described previously [48]. PSMs were quantified by integrating the ion chromatograms extracted by the mass-to-charge ratios of doubly and triply charged ions of each PSM. The unit of PSM measurement is “amount arbitrary unit (A.U.)”.

### 4.12. Western Blot for the Quantification of spa and hla Expression

For analysis of protein A and alpha-hemolysin expression, the isolates were cultured overnight and 10 µL of this culture was added to 1 mL TSB and incubated for 8 h at 37 °C. The cultures were centrifuged, and a 300-µL aliquot of the supernatant was used for Western blot analysis. Supernatants were submitted at 95 °C for 5 min and loaded in the 12% SDS page gel. The applied voltage was 150 v, 400 mA for 1 h. The proteins were transferred to a nitrocellulose membrane, washed, and blocked with Rabbit antibody for alpha-toxin. The membranes were audiographed in the Typhon TRIO plus Variable Mode Imager^®^. GraphPad Prism^®^ using a *t*-test analyzed the data.

### 4.13. Pulsed-Field Gel Electrophoresis (PFGE)

PFGE typing consisted of digestion with *Sma*I of chromosomal DNA from *S. aureus* isolates associated with VAP. The plug preparation protocol and parameters followed McDougal et al. [49]. The restriction fragments resulting from *Sma*I digestion were subjected to electrophoresis on 1% agarose gel in a CHEF-DR III system (Bio-Rad Laboratories, Hercules, CA, USA), with pulses alternating from 5 to 60 s at 6 V/cm and 13 °C for 23 h.

The gels were stained with Gel Red (Biotium, San Francisco, CA, USA) for 1 h, washed in Milli Q water for another hour, and photographed under UV transillumination. The profiles were analyzed with the BioNumerics software (Applied Maths, Sint-Martens-Latem, Belgium) using the Dice similarity coefficient and the UPGMA method (1.2 tolerance and 1% optimization) for cluster analysis of isolates. Clusters were defined when similarity was ≥80%. International clones were kindly provided by Antônio Carlos Campos Pignatari, Special Laboratory of Clinical Microbiology, Department of Infectology, Federal University of São Paulo-Escola Paulista de Medicina and Agnes Marie Sá Figueiredo, Paulo de Góes Institute of Microbiology, Federal University of Rio de Janeiro, Brazil.

### 4.14. Multilocus Sequence Typing (MLST)

The constitutive genes of *S. aureus* associated with VAP were amplified and sequenced to determine allele composition and sequence type (ST) using the parameters proposed by Enright et al. [50]. Seven housekeeping genes were used (*arc*C, *aro*E, *yqi*L, *gmk*, *tpi*, *glp*, and *pta*). Sequencing was performed using Macrogen (Rockville, MD, USA), and sequences were analyzed using SeqBuider^®^ and the official website mlst.org.

### 4.15. Statistical Analysis

Poisson regression of host and microbiological factors was performed to evaluate their relationship with the development of VAP and death, using backward selection (the first model included all variables, and the multivariate model included variables with *p* > 0.1, removing the variable with the highest *p*-value at each step and including only those with *p* < 0.1, i.e., significant, and marginally significant variables). The values obtained for the production of PSM, alpha-toxin, and protein A (spa) were dichotomized by the median for inclusion in the model (‘yes’ for a value equal to or greater than the median and ‘no’ for a lower value). Data were stored in Epi Info^®^ 3.5.2 (Centers for Disease Control and Prevention) and Excel and were analyzed using Epi Info and SPSS^®^. The incidence density of *S. aureus* and MRSA VAP was calculated using the following equation:

$$ID=\frac{NVAP\times 1000}{\Sigma nVis\times 3.5}$$

where ID = incidence density, NVAP = number of patients with VAP, 1000 = standard coefficient, ΣnVis = sum of the number of visits of patients (728), and 3.5 = interval between visits in days.

## 5. Conclusions

Our results allow us to suggest that in ICU patients, VAP is mainly caused by *S. aureus* strains that already colonize these patients. This suggestion is supported by the STs identified, which are characteristic of strains found in the community that produce important virulence factors involved in the development of VAP and subsequent death.

## Figures and Tables

**Table 1:** Determination of minimum inhibitory concentration in *S. aureus* using the E-test.

Antimicrobial	MIC Range	Susceptibility (%)	MIC50	MIC90
Oxacillin	0.016–>256	55	0.5	>256
Vancomycin	0.125–1.5	100	0.75	1.5
Quinupristin- Dalfopristin	0.094–0.75	100	0.38	0.75
Tigecycline	0.047–0.94	100	0.094	0.125
Linezolid	0.19–1.5	100	0.75	1
Daptomycin	0.016–0.47	100	0.094	0.125

MIC: minimum inhibitory concentration; MIC50: minimum inhibitory concentration that inhibits 50% of the isolated microorganisms; MIC90: minimum inhibitory concentration that inhibits 90% of the isolated microorganisms.

**Table 2:** Frequency of virulence genes and association with the presence of the *mec*A gene.

Virulence Genes	Total (%)	*mec*A		*p-*Value
		Positive N (%)	Negative N (%)	
*hla*	53 (100)	23 (100)	30 (100)	1
*hld*	53 (100)	23 (100)	30 (100)	1
*icaA*	53 (100)	23 (100)	30 (100)	1
*icaD*	44 (83)	19 (82)	25 (83)	0.08
*hlb*	18 (34)	9 (39)	9 (30)	0.48
*sea*	17 (32)	5 (21)	12 (10)	0.15
*sec*	10 (19)	5 (21)	5 (16)	0.21
*icaC*	6 (11)	4 (17)	2 (6)	0.61
*tst*	1 (2)	0	1 (3)	0.89
*pvl*	1 (2)	0	1 (3)	0.89
*eta*	0	0	0	--
*etb*	0	0	0	--
*seb*	0	0	0	--
*bap*	0	0	0	--

**Table 3:** Characteristics of *S. aureus* strains isolated from patients with ventilator-associated pneumonia.

Strain	ST	*mec*A	SCC*mec*	Virulence Genes	Alpha-Toxin (A.U)	Protein A (A.U)
47	105	+	II	*hla/hlb/hld/icaA/icaD/sea*	24373527	9767104
54	398	-		*hla/hld/icaA/icaD/sea*	76362660	0
64	1635	-		*hla/hlb/hld/icaA/icaD*	0	32265275
70	1	-		*hla/hlb/hld/sea/icaA/icaD*	6982199	21823765
97	398	-		*hla/hld/sea/sec/icaA/icaD*	41033870	455862
129	8	+	IV	*hla/hld/sea/icaA/icaD*	38447276	1576784
148	slv 546	-		*hla/hld/sec/icaA*	41687067	0
166-1	5	-		*hla/hld/sea/sec/icaA/icaD*	7356952	10672368
166-2	5	+	IV	*hla/hld/icaA*	0	0
211	5	+	IV	*hla/hld/icaA/icaD*	0	28944479
220-1	45	-		*hla/hlb/hld/sea/icaA/icaD*	0	0
220-2	45	-		*hla/hlb/hld/sea/icaA*	1939185	73614614
239	105	+	II	*hla/hlb/hld/icaA/icaD*	6919452	9918149

ST: sequence type; SLV: single locus variant; spa: protein A; A.U: unit of measurement is “Amount (Arbitrary Unit)”.

**Table 4:** Detection and quantification of phenol-soluble modulins (PSM) in *S. aureus* strains isolated from patients with ventilator-associated pneumonia.

Strain	PSM Alpha 1	PSM Alpha 2	PSM Alpha 3	PSM Alpha 3 N22Y	PSM Beta 1	PSM Beta 2	Delta Toxin	Delta Toxin G10S	PSM Delta *mec*
P47	641000000 *	228000000	240000000	__	237000000	72415500	2770000000	__	7590000000
54	1470000000	942000000	1510000000	__	218000000	__	10100000000	__	__
64	__ **	__	__	117000000	__	__	__		
70	470000000	212000000	194000000	__	101000000	62116000	1120000000	0	__
97	2120000000	1060000000	1430000000	__	265000000	83428420	8230000000	0	__
129	4430000000	2110000000	1900000000	__	286000000	99102590	7410000000	__	__
148	1150000000	640000000	438000000	__	143000000	__	9560001000	__	__
166-1	437000000	291000000	234000000	__	__	65587410	2220000000	__	__
166-2	__	__	__	107000000	__	__	__	__	__
211	__	__	__	101000000	__	__	__	__	__
220-1	2140000000	1470000000	1630000000	__	327000000	52123570	3740000000	0	__
220-2	__	__	__	__	__	__	__	__	__
239	647000000	325000000	294000000	__	189000000	66117540	2980000000	__	6860000000

* Unit of measurement is “Amount (Arbitrary Unit, A.U)”, ^____^** Below detection limit.

**Table 5:** Poisson regression model for analysis of the association of host and microbiological factors with the development of ventilator-associated pneumonia caused by *S. aureus*.

Risk Factors	Univariate	*p-*Value	Multivariate (Final Model)	*p-*Value
	RR (95% CI)		RR (95% CI)	
Category 1: demographic data				
Male gender	1.91 (0.68–5.30)	0.2		
Age	0.99 (0.98–1.02)	0.9		
Category 2: comorbidities				
Heart disease	1.30 (0.74–2.29)	0.4		
Lung disease	0.74 (0.27–2.07)	0.6		
Kidney disease	0.87 (0.41–1.85)	0.7		
Liver disease	2.28 (0.82–6.34)	0.1		
Central nervous system disease	**0.50 (0.26–0.97)**	**0.04**		
Solid tumor	1.99 (0.92–4.24)	0.08	**2.51 (1.08–5.88)**	**0.03**
AIDS	1.68 (0.61–4.69)	0.3		
Diabetes mellitus	1.16 (0.55–2.50)	0.7		
Trauma	0.81 (0.29–2.26)	0.7		
Category 3: procedures				
Indwelling urinary catheter	1.16 (0.42–3.25)	0.8		
Central venous catheter	0.69 (0.38–1.26)	0.2		
Surgery	0.74 (0.42–1.31)	0.3		
Use of steroids	0.52 (0.19–1.45)	0.2		
Category 4: antimicrobials				
Carbapenems	… *	… *		
Other beta-lactams (anti-pseudomonas)	1.05 (0.59–1.86)	0.9		
Other beta-lactams (non-anti-pseudomonas)	1.27 (0.71–2.25)	0.4		
Glycopeptides	0.58 (0.21–1.61)	0.3		
Quinolones	0.00 (0.00–…)	1		
Polymyxins	0.00 (0.00–…)	1		
Macrolides	1.39 (0.50–3.87)	0.5		
Sulfa drugs	… *	… *		
Anaerobicides	0.00 (0.00–…)	… *		
Category 5: resistance and virulence				
*mec*A	1.21 (0.69–2.14)	0.5		
SCC*mec*				
Absent (reference)	…	…		
*SCCmec I*	0.00 (0.00–…)	1		
*SCCmec II*	0.00 (0.00–…)	1		
*SCCmec III*	39.80 (0.00–…)	1		
*SCCmec IV*	200.04 (0.00–…)	1		
*pvl*	0.00 (0.00–…)	1		
*tsst-1*	0.00 (0.00–…)	1		
*hla*	4756.52 (0.00–…)	1		
*hlb*	1.25 (0.70–2.22)	0.4		
*hld*	0.95 (0.34–2.64)	0.9		
*etA*	… *	… *		
*etB*	… *	… *		
*sea*	**2.07 (1.17–3.69)**	**0.01**	**1.88 (1.04–3.41)**	**0.04**
*seb*	… *	… *		
*sec*	1.10 (0.57–2.11)	0.8		
*bap*	… *	… *		
*icaA*	… *	… *		
*icaC*	**1.92 (1.00–3.69)**	**0.049**		
*icaD*	10527 (0.00–…)	1		

RR: rate ratio; CI: confidence interval. * Analysis was not possible because of the same value in all subjects. The model was also adjusted for the median of phenol-soluble modulins, alpha-toxin, and protein A. Significant associations are indicated in bold.

**Table 6:** Poisson regression model for analysis of the association of host and microbiological factors with the death of patients due to ventilator-associated pneumonia caused by *S. aureus*.

Risk Factors	Univariate	*p-*Value	Multivariate (Final Model)	*p-*Value
	RR (95% CI)		RR (95% CI)	
Category 1: demographic data				
Male gender	5063 (0.00–…)	1		
Age	0.99 (0.96–1.03)	0.9		
Category 2: comorbidities				
Heart disease	1.10 (0.45–2.70)	0.8		
Lung disease	0.00 (0.00–…)	1		
Kidney disease	0.97 (0.33–2.92)	0.9		
Liver disease	**3.63 (1.21–10.85)**	**0.02**		
Central nervous system disease	0.48 (0.16–1.44)	0.2		
Solid tumor	0.00 (0.00–…)	1		
AIDS	2.66 (0.89–7.96)	0.08		
Diabetes mellitus	1.24 (0.41–3.71)	0.7		
Trauma	1.43 (0.48–4.28)	0.5		
Category 3: procedures				
Indwelling urinary catheter	0.66 (0.222–1.99)	0.5		
Central venous catheter	**0.39 (0.16–0.97)**	**0.04**		
Surgery	0.43 (0.14–1.29)	0.1		
Use of steroids	0.88 (0.30–2.65)	0.8		
Category 4: antimicrobials				
Carbapenems	… *	… *		
Other beta-lactams (anti-pseudomonas)	1.56 (0.64–3.18)	0.3		
Other beta-lactams (non-anti-pseudomonas)	0.51 (0.17–1.51)	0.2		
Glycopeptides	0.97 (0.33–2.92)	0.9		
Quinolones	0.00 (0.00–…)	1		
Polymyxins	0.00 (0.00–…)	1		
Macrolides	2.05 (0.69–6.14)	0.2		
Sulfa drugs	… *	… *		
Anaerobicides	0.00 (0.00–…)	… *		
Category 6: resistance and virulence				
*mec*A	0.92 (0.38–2.25)	0.9		
SCC*mec*				
*Absent (reference)*	…	…		
*SCCmec I*	0.00 (0.00–…)	1		
*SCCmec II*	4021.00 (0.00–…)	1		
*SCCmec III*	… *	… *		
*SCCmec IV*	0.00 (0.00–…)	1		
*pvl*	0.00 (0.00–…)	1		
*tsst-1*	0.00 (0.00–…)	1		
*hla*	2910.00 (0.00–…)	1		
*hlb*	1.89 (0.77–4.63)	0.2	**3.85 (1.43–10.32)**	**0.007**
*hld*	4188.26 (0.00–…)	1		
*etA*	… *	… *		
*etB*	… *	… *		
*sea*	**3.24 (1.08–9.67)**	**0.04**	**6.58 (1.86–23.38)**	**0.003**
*seb*	… *	… *		
*sec*	1.40 (0.51–3.42)	0.5		
*BAP*	… *	… *		
*icaA*	… *	… *		
*icaC*	1.62 (0.54–4.85)	0.4		
*icaD*	6358.39 (0.00–…)	1		

RR: rate ratio; CI: confidence interval. * Analysis was not possible because of the same value in all subjects. In the multivariate analysis, the model was adjusted considering the median value of phenol-soluble modulins, alpha-toxin, and protein A. Significant associations are indicated in bold.

**Table 7:** Detection of genes encoding virulence factors in *S. aureus*.

Genes	bp	Positive Control	References
*sea*	120	ATCC 13565	Johnson et al., Cunha et al. [41,42]
*seb*	478	ATCC 14458	Johnson et al., Cunha et al. [41,42]
*sec-1*	257	ATCC 19095	Johnson et al., Cunha et al. [41,42]
*tst*	350	ATCC 51650	Johnson et al., Cunha et al. [41,42]
*lukPV*	433	ATCC 49775	Lina et al. [16]
*icaA*	600	ATCC 35556	Rohde et al. [43]
*icaD*	450	ATCC 35556	Arciola et al. [44]
*Bap*	220	None	Cucarella et al. [45]
*eta*	119	ZM	Jarraud et al. [46]
*etb*	200	N5	Jarraud et al. [46]
*hla*	209	N315	Jarraud et al. [46]
*hlb*		RN4220	Jarraud et al. [46]
*hld*	357	ATCC 19095	Marconi et al. [47]

bp: base pairs.

## Data Availability

The data presented in this study are original and have not been published in scientific journals. The only document that contains these data is the doctoral thesis of Mariana Fávero Bonesso, openly available in [Institutional Repository of UNESP] at [http://hdl.handle.net/11449/139372] (accessed on 1 June 2023).

## Supplementary Materials

The following supporting information can be downloaded at https://www.mdpi.com/article/10.3390/antibiotics12081336/s1, Table S1. The data sheet with all the data for all 51 *S. aureus* strains isolated in the study.
